# Development and Application of a Composite Water-Retaining Agent for Ecological Restoration in Arid Mining Areas

**DOI:** 10.3390/polym17172268

**Published:** 2025-08-22

**Authors:** Liugen Zhang, Zhanwen Cao, Zhaojun Yang, Yi Zhang, Jia Guo

**Affiliations:** 1 Xinjiang Hami Santanghu Energy Development and Construction Co., Ltd., Hami 839000, China; xjnyzlg1122@126.com (L.Z.); 13919372459@139.com (Z.C.); 2 Key Laboratory of Oil and Gas, School of Chemical Engineering, Xinjiang University, Urumqi 830017, China; 3 College of Chemistry and Chemical Engineering, Central South University, Changsha 410083, China; 232311053@csu.edu.cn

**Keywords:** water-retaining agents, acrylic acid, sodium carboxymethyl cellulose, humic acids, rehabilitation of open-pit coal mines in arid areas

## Abstract

Ecological restoration in arid coal-mining regions faces extreme challenges due to soil infertility, salinization, and water scarcity. This study addresses these limitations in the Santanghu Shitoumei No. 1 open-pit mine (Xinjiang), where gypsum gray-brown desert soil, minimal rainfall (199 mm/yr), high evaporation (1716 mm/yr), and persistent gale-force winds exacerbate revegetation efforts. To overcome the high cost, short lifespan, and poor practicality of commercial water-retaining agents, we developed a novel humic acid (HA) and sodium carboxymethyl cellulose (CMC) composite water-absorbing resin (HA-CMC). Optimal synthesis parameters—identified as acrylic acid (AA)–carboxymethyl cellulose (CMC)–humic acid (HA)–Acrylamide (AM)–N,N’-methylene diacrylamide (MBA)–Ammonium persulphate (APS) = 100%:15%:4.5%:25%:0.6%:0.8%—yielded effective crosslinking, confirmed via FTIR and SEM. Performance benchmarking against existing agents demonstrated superior attributes. Field application in the mine’s demonstration area significantly enhanced surface vegetation and soil fertility, confirming the resin’s potential for large-scale soil remediation and ecological restoration in arid mining environments.

## 1. Introduction

Coal mining, while economically critical, causes irreversible damage to fragile ecosystems, particularly in regions rich in coal reserves. Xinjiang, China, is home to the nation’s largest coal deposits and open-pit mining operations, which have led to widespread loss of surface vegetation, soil degradation, and water pollution [1,2,3,4]. Traditional reclamation techniques often fail to address the dual challenges posed by extreme climates and Saline–alkali soil environment in arid regions. There is an urgent need to develop efficient, low-cost materials for environmental remediation [5]. Water-retaining agents stand out among environmental remediation materials owing to their outstanding water-holding capacity and nutrient retention properties, making them essential for both soil structure amelioration and vegetation rehabilitation. In particular, superabsorbent polymers (SAPs) offer potential through water retention and soil conditioning, yet current variants face three critical limitations: (1) performance degradation in saline–alkali soil environment, (2) complex synthesis, and (3) poor ecological compatibility. These shortcomings hinder their large-scale application in coal mining regions. There is a pressing need for a new type of water-retaining agent that offers high salt tolerance, strong water retention, and environmental sustainability.

The development of water-retaining agents has evolved from the toxic starch-grafted acrylonitrile polymers in the early 1960s [6,7,8,9] to the optimized polyacrylic acid-based materials in the early 2000s [10]. As a result, salt resistance and environmental compatibility have gradually improved [11,12,13,14]. However, unresolved challenges persist, limiting their real-world applicability. The difficulties are as follows: 1. Many water absorption performance tests typically use deionized water, which cannot reflect complex saline ion environments. 2. Conventional synthesis methods often rely on reverse-phase suspension techniques, which demand intensive post-treatments [15,16,17]. 3. Natural SAPs exhibit low absorption, while synthetics resist degradation and risk secondary pollution. Critically, salt tolerance often compromises water retention capacity. This study introduces a facile “humic acid–polyacrylic acid composite modification” strategy, offering innovations in both material design and application scenarios. To address the balance between salt tolerance, water retention and ecological compatibility, humic acid was chosen as the key additive owing to its abundant carboxyl and hydroxyl functional groups. This modification enables synergistic hydrogen bonding and ion crosslinking with polyacrylic acid, enhancing salt tolerance, gel stability, and soil fertility. A simplified one-step aqueous polymerization optimizes monomer ratios and crosslinking density, simplifying the synthesis process and reducing costs. Finally, Performance was quantified under realistic mining ion conditions, and a “material–soil–vegetation” restoration system was established via drought-resistant plant trials. By addressing the core constraints of arid mining restoration, we hope this composite polymer will offer a novel technical approach for rehabilitating degraded ecosystems.

## 2. Materials and Methods

### 2.1. Materials

Humic acid (HA, analytical pure, No. 809, Chuhua Branch Road, Fengxian District, Shanghai city, Shanghai Aladdin Biochemical Technology Co., Ltd., Shanghai, China), carboxymethyl cellulose sodium (CMC, analytical pure, Shanghai Aladdin Biochemical Technology Co., Ltd.), acrylamide (AM, analytical pure, Shanghai Aladdin Biochemical Technology Co., Ltd.), N, N-methylbisacrylamide (MBA, analytical pure, Shanghai Aladdin Biochemical Technology Co., Ltd.), Ammonium persulphate (APS, analytical pure, West of Yuejin Road, Li Mingzhuang, Huaming Street, Dongli District Tianjin city, Tianjin Zhiyuan Chemical Reagent Co., Ltd., Tianjin, China), sodium hydroxide (NaOH, Room 441, 4th Floor, Building B, No. 189 Xinda Road, Wanghaolou Street, Hebei District, Tianjin city, Tianjin Xinbote Chemical Co., Ltd., Tianjin, China). Sodium chloride (NaCl, analytical pure, Tianjin Zhiyuan Chemical Reagent Co., Ltd.). Other chemical reagents were analytical-grade and utilized as obtained without further purification.

### 2.2. Synthesis of Resin

The synthesis procedure involved neutralizing 5 g acrylic acid (70–80% degree) with 40 wt% NaOH in a 250 mL three-necked flask, followed by adding potassium persulfate initiator and N,N′-methylenebisacrylamide crosslinker, polymerizing at 70 °C for 3 h under N_2_ atmosphere, drying the resultant gel (which was then comminuted into granules) at 80 °C, and finally measuring water absorption of the dried product.

### 2.3. Swelling Performance

Measurement steps: Take resin with a mass of m (about 0.1 g) and place it at the bottom of a tea bag. Disperse it evenly. Seal the tea bag. Soak it in a sufficient amount of 0.9% sodium chloride solution, distilled water, or irrigation water for mining areas. Let it stand until the SAP absorbs the liquid and swells completely. Take out the tea bag and let it stand until there are no free water droplets. Weigh the resin mass and calculate the swelling rate, and weight (m_1_). Use tea bags without samples for blank comparison, and weigh the mass of the blank experimental tea bag as m_2_.

Calculation result: The water retention capacity R of resin is calculated according to the following formula:

R(g/g) = (m_1_ − m_2_)/m

In the formula, m is the mass of the resin sample, m_1_ is the mass of the tea bag containing resin, and m_2_ is the mass of the blank experimental tea bag.

### 2.4. Repeated Water Absorption Performance

A measured quantity of resin was used to evaluate its repeated water absorption capacity. Following the water absorption ratio testing method described in Section 2.3., the resin sample that had completed one absorption cycle was dried in an oven. The dried sample was then subjected to a second absorption test. This process was repeated multiple times to determine the resin’s repeated water absorption performance.

### 2.5. Water Absorption Rate and Kinetic Study

An appropriate amount of the sample was placed in a tea bag and immersed in deionized water. The tea bag was removed at specific time intervals (1, 3, 5, 10, 20, 30, 60, 120, 180, and 300 min), and surface moisture was blotted using filter paper before weighing. The water absorption rate was then calculated. A trend graph illustrating the water absorption kinetics of the resin was subsequently plotted.

### 2.6. Salt Tolerance Performance

Salt solutions of sodium chloride (NaCl), calcium chloride (CaCl_2_), and ferric chloride (FeCl_3_) were prepared at concentrations of 0.01, 0.03, 0.05, 0.1, and 0.15 mol/L, respectively. The water-absorbing resin was then immersed in these salt solutions of varying concentrations, and its water absorption capacity was recorded.

### 2.7. Characteristics of Resin

SEM Analysis: The resin was swollen to 2–3 times its original volume, rapidly frozen with liquid nitrogen, and freeze-dried for 24 h. The micro-morphology was then observed and recorded using a field-emission scanning electron microscope (JSM-7001F, Jeol Ltd., Musashino, Akishima City, Tokyo, Japan).

FT-IR Analysis: The resin powder was mixed with KBr and pressed into pellets, then scanned and recorded using a VERTEX 70 RAMI spectrometer (Bruker Technology Co., Ltd., Saarbrücken, Germany).

XRD Analysis: X-ray diffraction patterns were recorded using a D8 advance diffractometer with a scanning range of 2θ = 5–80° at a rate of 5°/min (Bruker Technology Co., Ltd., Saarbrücken, Germany).

TG Analysis: Thermogravimetric analysis was performed using a HITACHI STA7300 analyzer under a nitrogen atmosphere, with temperature ramping from room temperature to 600 °C to record weight loss behavior (Hitachi Ltd., Tokyo, Japan).

## 3. Results and Discussion

### 3.1. Optimization of the Optimal Ratio of Each Material and Reaction System

This study systematically investigated the key parameters influencing the swelling performance of sodium carboxymethylcellulose–acrylic acid–polyacrylamide–humic acid (CMC-AA-AM-HA) composite water-retaining agent. Six critical factors were examined: (1) reaction temperature, (2) acrylic acid neutralization degree, (3) N,N’-methylenebisacrylamide (MBA) crosslinker concentration, (4) ammonium persulfate (APS) initiator dosage, (5) humic acid (HA) content, and (6) AM dosage. Through controlled experiments, we identified optimal conditions that maximize the water absorption capacity while maintaining desirable structural integrity of the superabsorbent polymer network.

#### 3.1.1. Effect of Temperature on the Water Absorption Rate of Superabsorbent Resins

**Figure 1:**
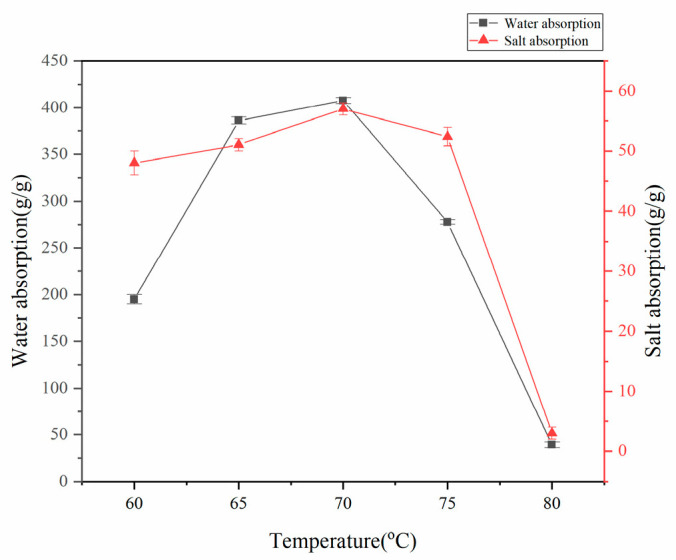
Effect of reaction temperature on water absorption rate of absorbent resin.

Under controlled synthesis conditions (75% acrylic acid neutralization, mass ratios w(AA):w(CMC):w(HA):w(AM):w(MBA):w(APS) = 100:15:4.5:25:0.6:0.8), the water absorption capacity demonstrated a temperature-dependent optimum, peaking at 70 °C (Figure 1). Below 65 °C, reduced initiator activity led to insufficient radical generation and slow polymerization, yielding weakly crosslinked networks with increased soluble fractions and poor mechanical strength. The 70 °C optimum balanced radical production and reaction kinetics, forming an effective three-dimensional network. However, temperatures exceeding 70 °C caused (1) excessive initiator decomposition rates producing low-molecular-weight polymers with shortened backbones, (2) decreased crosslinking density, and (3) potential thermal runaway due to accelerated exothermic polymerization, ultimately resulting in viscous, low-strength products with compromised water absorption performance.

#### 3.1.2. Effect of MBA on the Water Absorption Rate of Superabsorbent Resins

**Figure 2:**
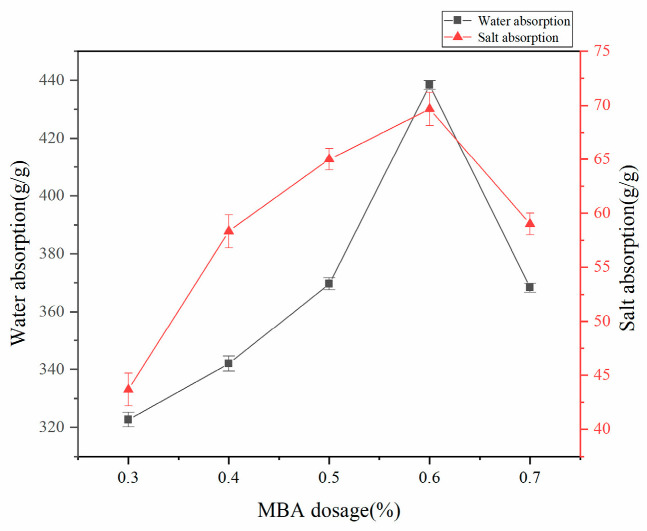
Effect of MBA dosage on water absorption rate of absorbent resin.

Under controlled synthesis conditions (75% acrylic acid neutralization, 70 °C reaction temperature, and mass ratios w(AA):w(CMC):w(HA):w(AM):w(APS) = 100:15:4.5:25:0.8), the water absorption performance exhibited a non-monotonic dependence on MBA crosslinker content, reaching maximum absorption at 0.6% MBA dosage (Figure 2). Superabsorbent polymers (SAPs), as functional polymeric materials with moderately crosslinked structures, demonstrate a significant correlation between swelling performance and crosslinking network density. Research indicates that optimal control of crosslinker content is crucial: insufficient crosslinking fails to establish a complete three-dimensional crosslinked network structure, leading to increased soluble component ratios and consequently reduced water absorption capacity, whereas excessive crosslinking significantly enhances the rigidity of the polymer network, not only constraining the elastic deformation capacity of molecular chains but also substantially diminishing the hydration space volume of the network structure, ultimately resulting in decreased water absorption performance of the resin.

#### 3.1.3. Effect of APS Dosage on Water Absorption of Superabsorbent Resins

**Figure 3:**
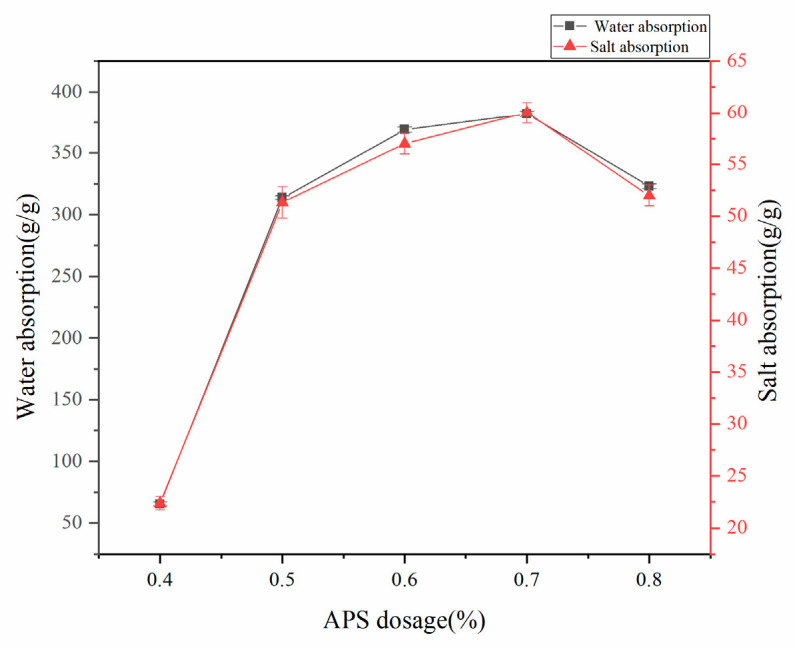
Effect of APS dosage on water absorption rate of absorbent resin.

Under controlled synthesis conditions (75% acrylic acid neutralization, 70 °C reaction temperature, and mass ratios w(AA):w(CMC):w(HA):w(AM):w(MBA) = 100:15:4.5:25:0.6), the water absorption capacity demonstrated a pronounced dependence on APS initiator dosage, exhibiting an initial increase followed by a decrease (Figure 3). This optimal behavior arises from competing radical-mediated mechanisms: (1) at low APS concentrations (<0.8%), insufficient radical generation leads to incomplete network formation and increased soluble fractions, while (2) excessive APS (>0.8%) produces radical overpopulation that accelerates termination reactions, reducing polymer molecular weight and creating excessive crosslinking that constrains the three-dimensional network architecture. The balanced condition (0.8% APS) achieves the optimal radical concentration for forming an effective porous network that maximizes water absorption.

#### 3.1.4. Effect of Neutralization Degree on Water Absorption Rate of Resin

**Figure 4:**
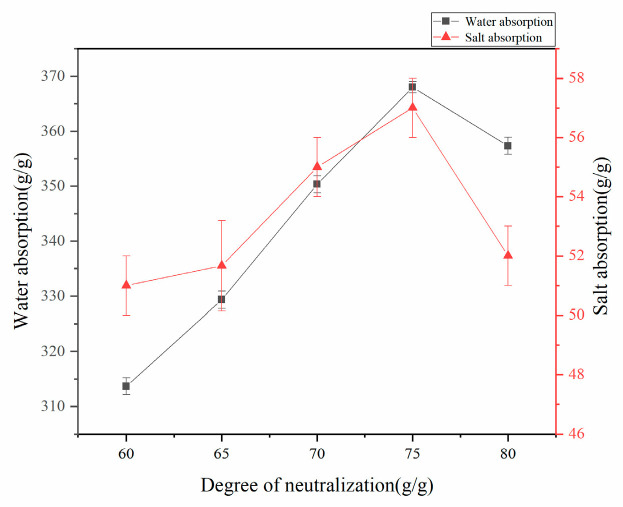
Effect of acrylic acid neutralization on water absorption of resins.

Under controlled reaction conditions (70 °C, mass ratios w(AA):w(CMC):w(HA):w(AM):w(MBA):w(APS) = 100:15:4.5:25:0.6:0.8), the influence of neutralization degree on the water absorption capacity of superabsorbent resin was systematically investigated. As presented in Figure 4, the water absorption performance exhibited a distinct maximum at 75% neutralization degree, demonstrating an initial increase followed by a gradual decrease with further neutralization.

This optimal behavior can be explained by two competing mechanisms: At low neutralization degrees (<75%), the high reactivity of acrylic acid monomers leads to uncontrolled copolymerization and homopolymerization tendencies, while the insufficient ionic groups result in low osmotic pressure and, consequently, reduced water absorption. When excessive neutralization occurs (>75%), the decreased reactivity of sodium acrylate (CH_2_=CHCOONa) compared to acrylic acid (CH_2_=CHCOOH) slows down the grafting reaction rate and reduces crosslinking density, ultimately increasing the soluble fraction and compromising the water absorption capacity.

#### 3.1.5. Effect of HA Dosage on Water Absorption of Superabsorbent Resin

**Figure 5:**
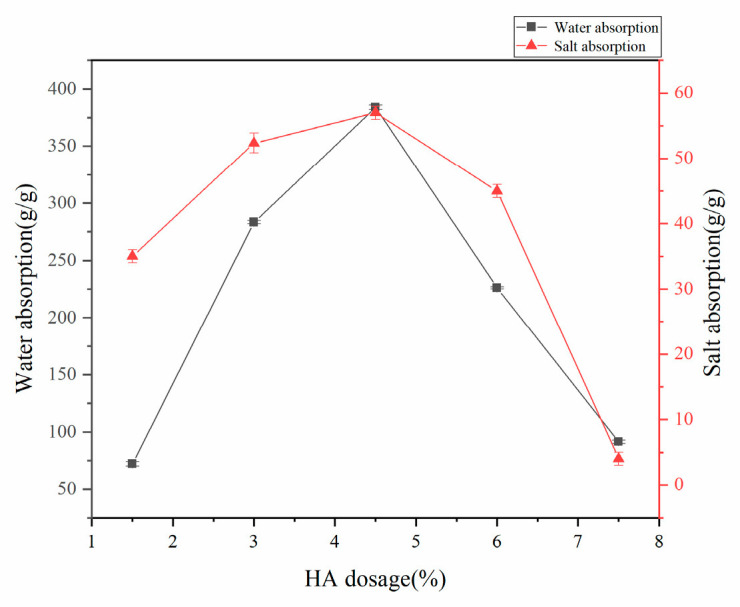
Effect of HA dosage on water absorption rate of superabsorbent resin.

Under controlled synthesis conditions (75% neutralization degree of acrylic acid, 70 °C reaction temperature, mass ratios w(AA):w(CMC):w(AM):w(MBA):w(APS) = 100:15:25:0.6:0.8), the influence of HA content on the water absorption properties of superabsorbent resin was systematically evaluated. As depicted in Figure 5, the water absorption capacity demonstrated a characteristic initial enhancement followed by a subsequent reduction as the HA dosage increased. This non-monotonic behavior originates from the dual functionality of HA: at optimal concentrations, its abundant carboxyl, hydroxyl, and amino groups participate in crosslinking reactions with AM and CMC to enhance network performance, whereas excessive HA leads to incomplete dissolution and physical entrapment within the polymer matrix, consequently disrupting osmotic equilibrium and diminishing absorption efficiency.

#### 3.1.6. Effect of AM Dosage on Water Absorption Rate of Superabsorbent Resins

**Figure 6:**
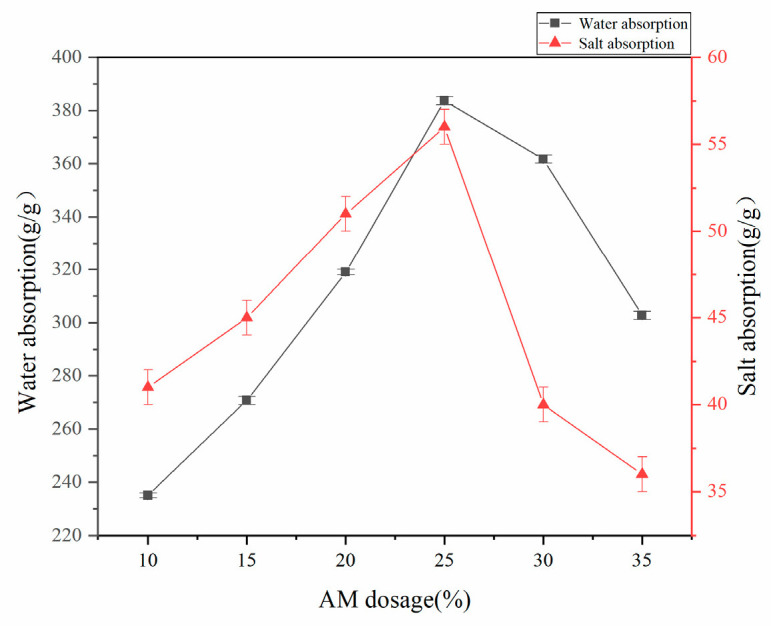
Effect of AM dosage on water absorption of superabsorbent resin.

Under controlled synthesis conditions (75% acrylic acid neutralization degree, 70 °C reaction temperature, mass ratios of w(AA):w(CMC):w(MBA):w(APS) = 100%:15%:0.6%:0.8%), the influence of AM dosage on the water absorption capacity of the superabsorbent resin was systematically investigated. As illustrated in Figure 6, the resin’s water absorption exhibited a distinct volcano-shaped trend with increasing AM content, reaching maximum absorption at 25% AM loading. This optimal performance arises from the synergistic effects between AM’s nonionic -CONH_2_ groups and the existing -COOH/-OH groups, which collectively enhance hydrophilicity. However, excessive AM content (>25%) leads to reduced absorption capacity because (1) the -CONH_2_ group demonstrates inferior water affinity compared to -COOH, and (2) the hydrophobic character of -CONH_2_ becomes dominant at higher concentrations, thereby compromising the overall water absorption performance.

### 3.2. Correlation Characterization Analysis

#### 3.2.1. The Water-Holding Properties of the Resin

**Figure 7:**
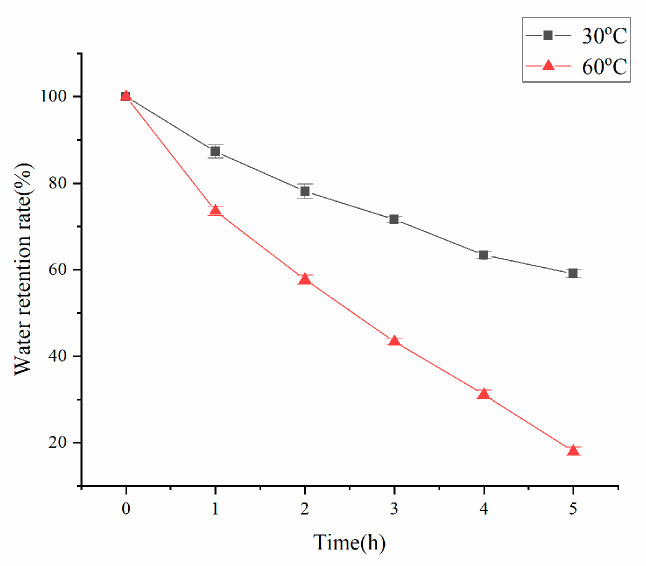
Water retention of resin at different temperatures.

Figure 7 demonstrates that the water retention capacity of the sample at absorption saturation exhibits temperature-dependent variations. Specifically, the water-holding performance showed significant differences when exposed to varying thermal conditions, indicating that the material possesses thermoresponsive characteristics.

The experimental results demonstrated a dramatic decrease in water retention capacity from 59.2% at 30 °C to merely 19% at 60 °C over 5 h. This thermal response indicates that (1) the resin’s crosslinked network maintains superior structural stability under low-temperature conditions while (2) retaining measurable water adsorption capacity even at elevated temperatures. Comparative analysis revealed substantially faster water evaporation rates at 60 °C versus 30 °C, and this was attributable to two primary mechanisms: (i) enhanced molecular thermal motion facilitating free water evaporation, and (ii) disruption of hydrogen bonding networks that normally stabilize water molecules within the polymer matrix.

#### 3.2.2. Swelling Kinetics of Resin in Water

**Figure 8:**
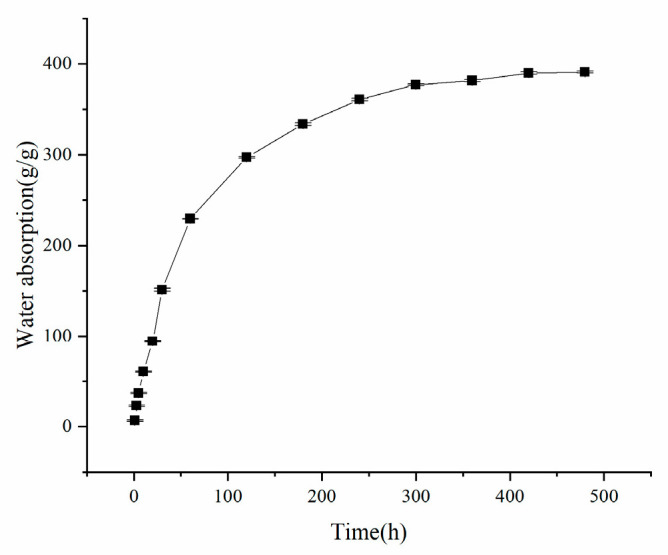
Resin water absorption rate graph.

Figure 8 during the initial hydration phase, the resin demonstrated rapid water absorption with a substantial swelling rate. The absorption kinetics exhibited distinct time-dependence: while the swelling rate was initially fast, it progressively decelerated over time, reaching near-equilibrium conditions at approximately 300 min.

#### 3.2.3. Repetitive Liquid Absorption Properties of Resins

**Figure 9:**
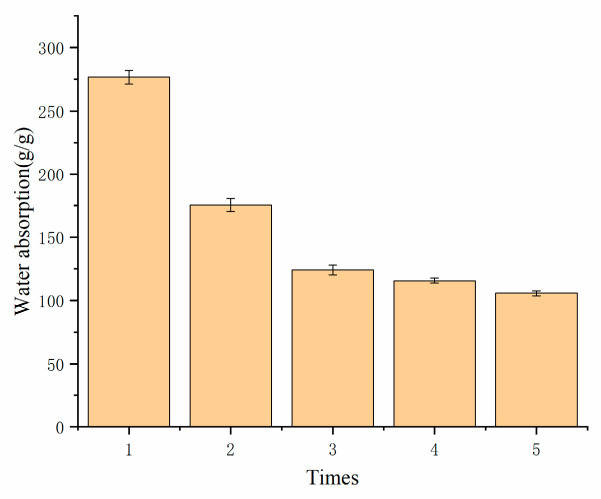
Reusability of resins.

Figure 9 after five absorption–desorption cycles, the resin exhibited a significant decline in water absorption capacity. This deterioration primarily stems from progressive damage to the crosslinked network during repeated hydration–dehydration processes, manifested through two mechanisms: (1) mechanical degradation of the crosslinked structure compromising three-dimensional network integrity, and (2) weakened elastic recovery of polymer chains, impairing the resin’s ability to maintain its original swollen state. These cumulative structural modifications collectively reduced the resin’s water retention capacity and absorption efficiency by >40% compared to its initial performance.

#### 3.2.4. Water Absorption in Different Salt Solutions

In the selection of metal ions, sodium chloride (NaCl) serves as the primary component of soil salinization in arid mining areas and is commonly used to simulate saline–alkali environments. Calcium chloride (CaCl_2_) represents ubiquitous hardness ions in soil, while ferric chloride (FeCl_3_) was chosen to investigate the unique effects of high-valence ions (with iron-rich wastewater frequently occurring in acid mine drainage). Therefore, these three ions were selected for testing.

**Figure 10:**
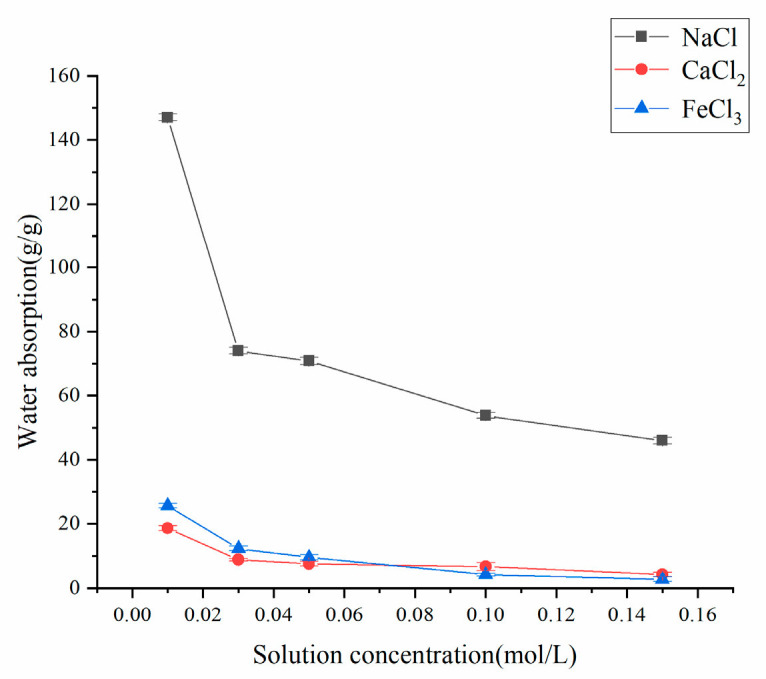
Water absorption properties of resins in different salt solutions.

Figure 10 demonstrates that the resin’s water absorption capacity in various salt solutions exhibited significant concentration-dependent behavior. The experimental results revealed a marked decrease in both equilibrium swelling and water absorption capacity with increasing salt concentration. This trend was most pronounced in NaCl solution, where the absorption rate dramatically declined from 148 g/g to 46 g/g as the NaCl concentration increased from 0.01 mol/L to 0.15 mol/L. The swelling inhibition mechanism primarily involves (1) reduced osmotic pressure gradient across the resin network, and (2) complexation of high-valence metal ions with carboxylic acid groups, leading to network contraction and a consequent reduction in liquid retention capacity.

Furthermore, the resin’s absorption performance showed an inverse relationship with metal ion valence. At equivalent concentrations, the absorption capacity followed the order NaCl > CaCl_2_ > FeCl_3_. This phenomenon arose because higher-valence cations (e.g., Ca^2+^ and Fe^3+^) exhibit stronger charge screening effects, more effectively neutralizing ionized groups within the resin and thus suppressing its swelling capacity.

This study elucidates that resin water absorption in saline environments is governed by multiple interrelated factors, including solution concentration, resin chemical architecture, and metal ion properties. A comprehensive understanding of these mechanisms is crucial for optimizing resin applications in wastewater treatment, ion exchange processes, and membrane separation technologies.

#### 3.2.5. SEM Topography Analysis

**Figure 11:**
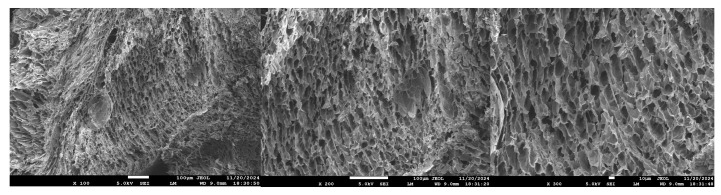
SEM images of composite resin at different magnifications.

Scanning electron microscopy (SEM) (Figure 11) analysis revealed that the sample surface exhibited a dense, irregular network structure with numerous pores. Following the CMC grafting reaction, the resin surface developed a distinct layered morphology, which facilitated the penetration of water molecules and enhanced the material’s water absorption capacity. This unique porous and layered structure not only increased the resin’s water absorption rate but also endowed the material with excellent liquid absorption properties.

#### 3.2.6. FTIR Analysis

**Figure 12:**
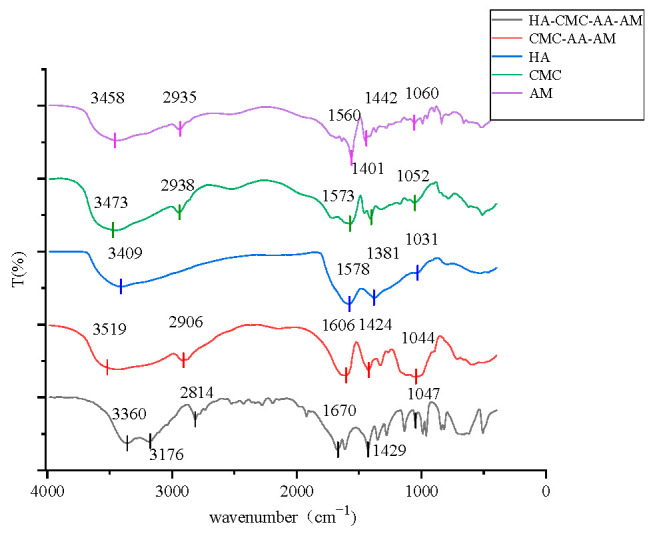
FTIR diagram of composite resin.

The structure of the composite resin was characterized by FT-IR spectroscopy (Figure 12). The spectrum exhibited distinct absorption peaks corresponding to key functional groups: a peak at 1047 cm^−1^ (C–O–C stretching vibration), 3360 cm^−1^ (–OH stretching vibration), 2814 cm^−1^ (symmetric/asymmetric –CH_2_ stretching vibrations), and 1429 cm^−1^ (COO^−^ symmetric stretching vibration), confirming the successful incorporation of HA into the resin matrix. Additionally, the peaks at 1670 cm^−1^ (–C=O stretching) and 3176 cm^−1^ (–NH_2_ stretching) corresponded to amide bonds, though their significantly reduced intensity indicated the successful introduction of AM.

Collectively, the presence of characteristic peaks associated with HA, PAA, and AM in the FT-IR spectrum confirmed the successful synthesis of the composite CMC-AA-AM/HA resin.

#### 3.2.7. TG and DTG Analysis

**Figure 13:**
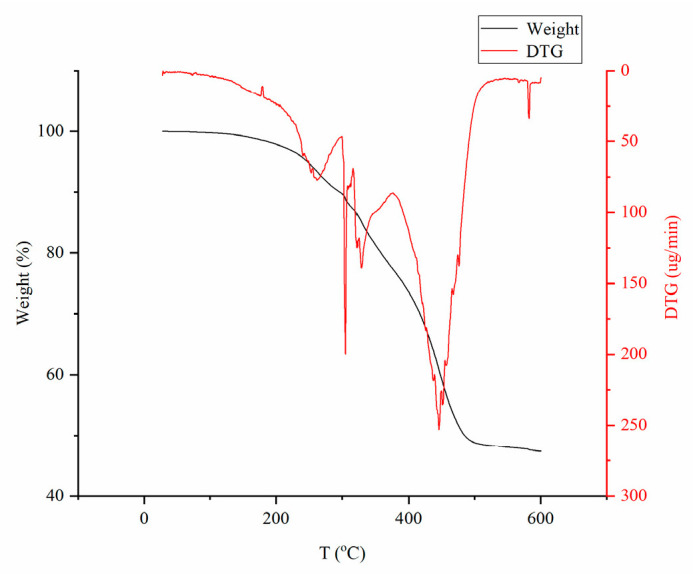
Thermogravimetric diagram of composite resin.

The TG and DTG curves of the composite resin (Figure 13) revealed a four-stage thermal degradation process. The initial weight loss corresponded to the evaporation of absorbed water, followed by the cleavage of amide bonds in AM during the second stage. The third stage involved thermal decomposition of glucoside units and scission of C-O bonds. In the final stage, weight loss occurred through three simultaneous pathways: (1) elimination of water molecules via anhydride formation between adjacent carboxyl groups on the polymer chain, (2) dehydration of sugar rings accompanied by C-O-C bond cleavage in the cellulose chain, and (3) breakdown of PAA chains and the crosslinked network structure.

#### 3.2.8. XRD Analysis

**Figure 14:**
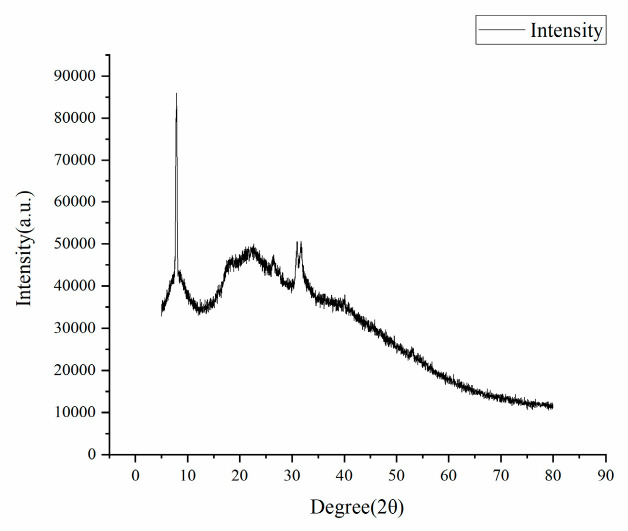
XRD plot of composite resin.

XRD analysis (Figure 14) of the composite resin surface revealed that the material retained a strong diffraction peak, demonstrating the preservation of its polymeric crystal structure and maintaining well-defined crystallinity.

#### 3.2.9. The Potential Mechanism of HA-CMC Interactions with Soil–Plant System

The incorporation of resin significantly improves soil’s structure by increasing porosity and enhancing permeability. As the SAP content increases, a notable reduction in soil bulk density is observed, attributable to the swollen resin forming a porous matrix that expands soil pore spaces. This structural modification not only facilitates gas exchange but also stimulates microbial activity, consequently elevating soil organic matter content. Furthermore, the HA component in HA-CMC demonstrates a sustained-release behavior during swelling, gradually releasing humic acid into the soil matrix. This dual function enhances soil fertility through continuous organic matter supplementation while simultaneously improving both physical (e.g., aeration, water retention) and chemical (e.g., nutrient availability) properties of the soil, thereby creating an optimized growth environment for plants. The slow-release mechanism ensures long-term nutrient supply for plant development while maintaining improved soil physicochemical characteristics [18].

## 4. Conclusions

In this study, we introduce a facile method to develop a humic acid–carboxymethyl cellulose composite superabsorbent polymer (HA-CMC) for ecological restoration in arid coal-mining regions. Integration of humic acid (HA) enabled both hydrogen bonding and ion crosslinking with the polyacrylic acid network, significantly enhancing salt tolerance (retaining > 90% water absorption in 0.9 wt% NaCl solution), gel stability, and soil organic content. A one-step aqueous polymerization process with optimized monomer ratios simplified production, reduced costs, and ensured scalability. Under real mining-area conditions, HA-CMC demonstrated excellent water retention capacity, boosting surface vegetation coverage by 40–60%, establishing a functional “material–soil–vegetation” restoration system. These results confirm that HA-CMC is a transformative solution for sustainable mining remediation, effectively resolving the trade-offs between salt tolerance, water retention, cost, and eco-compatibility that plague existing technologies. Future work will focus on large-scale deployment and long-term soil health monitoring.

## Data Availability

The original contributions presented in this study are included in the article/Supplementary Materials. Further inquiries can be directed to the corresponding authors.

## Supplementary Materials

The following supporting information can be downloaded at https://www.mdpi.com/article/10.3390/polym17172268/s1, Figure S1: pictures of the surface characteristics of bare rock gravel area; Figure S2 Plant growth at 10 days; Figure S3 Plant growth at 20 days; Figure S4 Plant growth at 45 days; Figure S5 Plant growth at 120 days; Table S1: Raw material cost (per ton of finished product); Table S2. Analysis of soil physical and chemical properties and evaluation of fertility indices.
