# Supplementary material for: Development and Application of a Composite Water-Retaining Agent for Ecological Restoration in Arid Mining Areas
Source: Polymers (Basel). 2025 Aug 22;17(17):2268. doi: 10.3390/polym17172268 (PMC12431388; doi:10.3390/polym17172268)
Supplement: Supplementary file 1 [file polymers-17-02268-s001.zip › polymers-3805331-supplementary.pdf]

Article

Development and Application of a Composite Water-Retaining Agent for Ecological Restoration in Arid Mining Areas

Zhang Liugen[1,3] ,Cao Zhanwen[1], Yang Zhaojun[2] ,Zhang Yi[2,3,*]， Guo Jia[1,*]

[1] Xinjiang Hami Santanghu Energy Development and Construction Co., Ltd; xjnyzlg1122@126.com
[2] College of Chemistry and Chemical Engineering, Central South University, Changsha 410083, P. R. China ; 232311053@csu.edu.cn
[3] Key Laboratory of Oil and Gas, School of Chemical Engineering，Xinjiang University, Urumqi 830017 Xinjiang; yzhangcsu@csu.edu.cn

* Correspondence: gj459824057@126.com; yzhangcsu@csu.edu.cn

This file includes

Feasibility Assessment and Cost-Benefit Analysis of Industrial Production:

From a technical perspective, the aqueous polymerization process demonstrates compatibility with existing superabsorbent polymer (SAP) production lines[1], ensuri ng straightforward industrial adaptation. Economically, the optimized formulation—incorporating cost-effective humic acid and mineral fillers—achieves reduction in ra w material costs compared to conventional SAPs. The supply chain exhibits robust scalability, with acrylic acid (AA) and acrylamide (AM) available through major ch emical suppliers (e.g., Sinopec), carboxymethyl cellulose (CMC) sourced from specia lized domestic manufacturers, and humic acid (HA) procurable directly from coal-ri ch regions. This integrated production system combines cost efficiency with scalabil ity advantages, making it viable for large-scale applications.

Table S1. Raw material cost (per ton of finished product)

	Material unit price	Composition proportion	Cost proportion
Acrylic acid（AA）	¥8.5/kg	68.8%	5848
carboxymethyl cellulose （CMC）	¥18/kg	10.3%	1854
Acrylamide（AM）	¥12/kg	17.2%	2064
humic acid（HA）	¥7/kg	3.1%	217
Sum total			¥9,983/ton

The cost reduction in this solution primarily stems from the substitution with humic acid (HA) and mineral fillers.

References

Academic Editor: Jesús-María García-Martínez

Received: 23 July 2025
Revised: 8 August 2025
Accepted: 14 August 2025
Published: 18 August 2025

Citation: Zhang, L.; Cao, Z.; Yang, Z.; Zhang, Y.; Guo, J. Development and Application of a Composite Water-Retaining Agent for Ecological Restoration in Arid Mining Areas. *Polymers* **2025**, *17*, x. https://doi.org/10.3390/xxxxx

Copyright: © 2025 by the authors. Submitted for possible open access publication under the terms and conditions of the Creative Commons Attribution (CC BY) license (https://creativecommons.org/licenses/by/4.0/).

1. Shandong Hist Biological Technology Co., Ltd. Production process of superabsorbent polymer for hygiene products: CN202411475630.4[P]. 2025-01-24. [in Chinese].

Experimental section
Figure S1. Pictures of the surface characteristics of bare rock gravel area.
Table S1. Analysis of soil physical and chemical properties and evaluation of fertility indices
Figure S2-5. Pictures of Plant growth in the mining area.

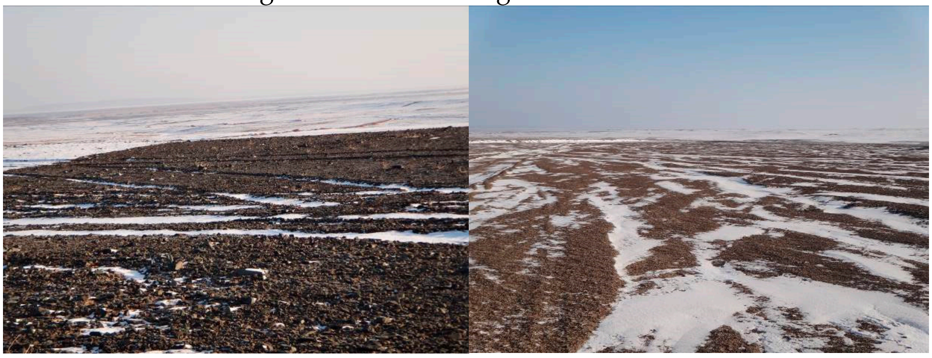

Fig. S1: pictures of the surface characteristics of bare rock gravel area

Table S2. Analysis of soil physical and chemical properties and evaluation of fertility indices

	pH	Salt content（%）	Total nitrogen（mg/kg）	Available nitrogen（mg/kg）	Total phosphorus（%）	Available phosphorus（mg/kg）	organic matter（g/kg）	Title 3
**Landfill soil**	8.9	9.3	0.30	31	0.17	15	3.25	
**Habitat soil**	8.4	4.2	0.36	32	0.18	16	3.60	
**Evaluate**	Heavily			Both types of soils are extremely poor				data

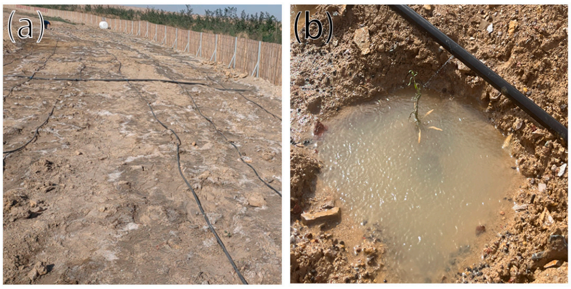

Fig. S2 Plant growth at 10 days

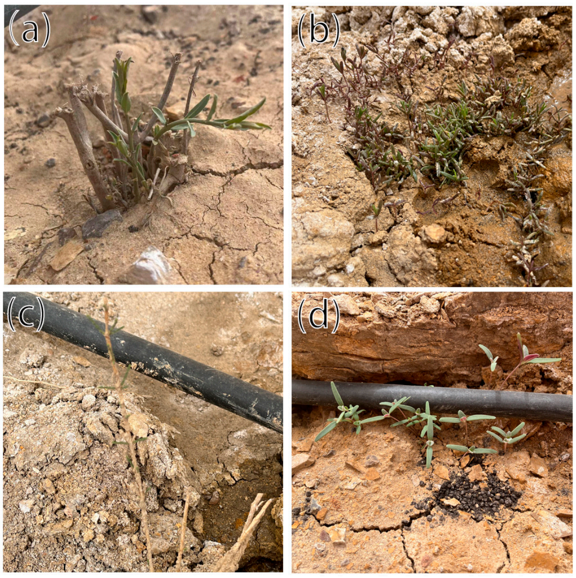

Fig. S3 Plant growth at 20 days

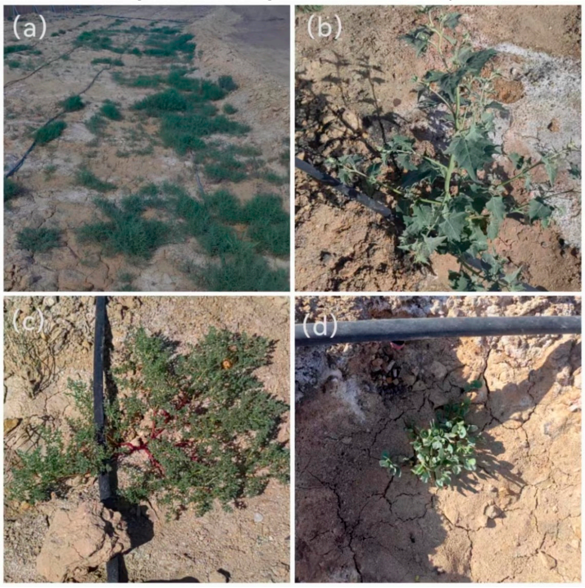

Fig. S4 Plant growth at 45 days

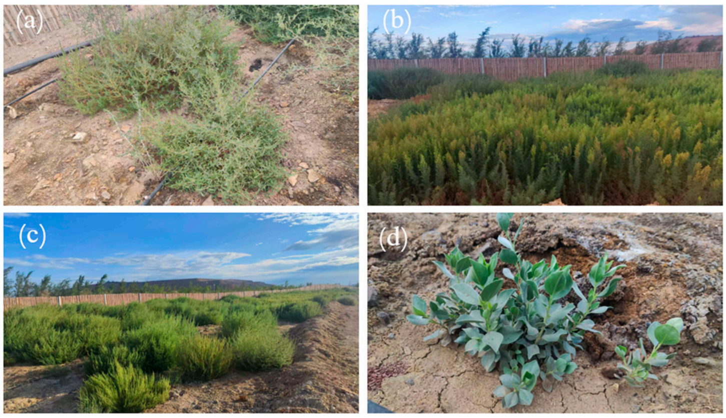

Fig. S5 Plant growth at 120 days
